# Metabolic Characterization of Peripheral Host Responses to Drainage-Resistant Klebsiella pneumoniae Liver Abscesses by Serum 1H-NMR Spectroscopy

**DOI:** 10.3389/fcimb.2018.00174

**Published:** 2018-06-01

**Authors:** Zhihui Chang, Hairui Wang, Beibei Li, Zhaoyu Liu, Jiahe Zheng

**Affiliations:** Department of Radiology, Shengjing Hospital of China Medical University, Shenyang, China

**Keywords:** metabolite, drainage-resistant, Klebsiella pneumoniae, liver abscess, 1H-nuclear magnetic resonance spectroscopy

## Abstract

**Purpose:** To explore the metabolic characterization of host responses to drainage-resistant Klebsiella pneumoniae liver abscesses (DRKPLAs) with serum 1H-nuclear magnetic resonance (NMR) spectroscopy.

**Materials and Methods:** The hospital records of all patients with a diagnosis of a liver abscess between June 2015 and December 2016 were retrieved from an electronic hospital database. Eighty-six patients with *Klebsiella pneumoniae* (*K. pneumoniae*) liver abscesses who underwent percutaneous drainage were identified. Twenty patients with confirmed DRKPLAs were studied. Moreover, we identified 20 consecutive patients with drainage-sensitive Klebsiella pneumoniae liver abscesses (DSKPLAs) as controls. Serum samples from the two groups were analyzed with 1H NMR spectroscopy. Partial least squares discriminant analysis (PLS-DA) was used to perform 1H NMR metabolic profiling. Metabolites were identified using the Human Metabolome Database, and pathway analysis was performed with MetaboAnalyst 3.0.

**Results:** The PLS-DA test was able to discriminate between the two groups. Five key metabolites that contributed to their discrimination were identified. Glucose, lactate, and 3-hydroxybutyrate were found to be upregulated in DRKPLAs, whereas glutamine and alanine were downregulated compared with the DSKPLAs. Pathway analysis indicated that amino acid metabolisms were significantly different between the DRKPLAs and the DSKPLAs. The D-glutamine and D-glutamate metabolisms exhibited the greatest influences.

**Conclusions:** The five key metabolites identified in our study may be potential targets for guiding novel therapeutics of DRKPLAs and are worthy of additional investigation.

## Introduction

Pyogenic liver abscess (PLA) is a life-threatening intra-abdominal infectious disease. In the two most recent decades, Klebsiella pneumoniae (*K. pneumoniae*) has become the most common causative pathogen of PLA in Asian countries as well as worldwide (Braiteh and Golden, 2007; Abate et al., 2012; Siu et al., 2012; Luo et al., 2014).

**Figure 1:**
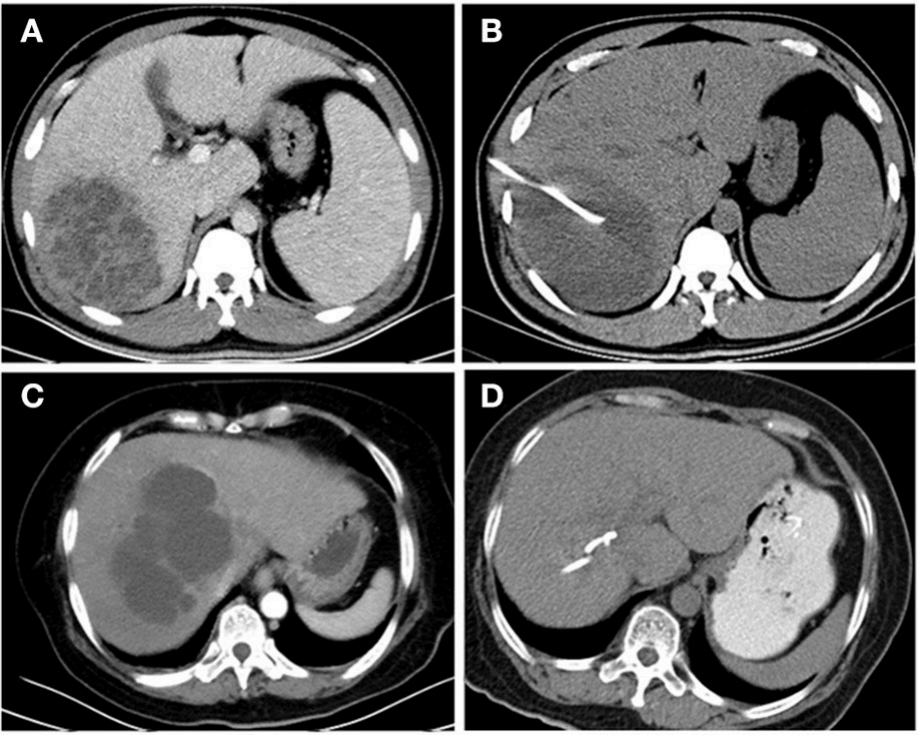
CT images of the livers of *K. pneumoniae* liver abscess patients before and 1 week after drainage. A 36-year-old man with a *K. pneumoniae* liver abscess **(A)**. The diameter of the abscess did not decrease 1 week after drainage **(B)**, and the abscess was thus defined as a drainage-resistant *K. pneumoniae* liver abscess (DRKPLA). A 62-year-old woman with a *K. pneumoniae* liver abscess **(C)**. The abscess had almost disappeared 1 week after drainage **(D)** and was thus defined as a drainage-sensitive *K. pneumoniae* liver abscess (DSKPLA).

The imaging features of *K. pneumoniae* liver abscesses (KPLAs) are similar to those of other types of liver abscesses. However, there are some notable differences. KPLAs more frequently appear as solid or multiloculated liver abscesses (Hui et al., 2007; Alsaif et al., 2011; Lee et al., 2011; Wang et al., 2014). Because of these features, some KPLAs are resistant to drainage because of the failure of liquefaction and require surgical intervention (Alkofer et al., 2012; Tan et al., 2013; Lo et al., 2015). Hence, it is of great importance to explore the host response mechanisms and develop treatment strategies to promote the liquefaction of KPLAs. However, to our knowledge, there are no reports that have explored the mechanism related to the failure of liquefaction of KPLAs. The failure of the treatment of a liver abscess by percutaneous drainage defines drainage-resistant liver abscesses (De Jong et al., 2010). In this study, we focused on drainage-resistant Klebsiella pneumoniae liver abscesses (DRKPLAs), and they had the following features: (a) the maximum diameter of the abscess did not decrease 1 week after drainage, and the drainage tube position was located within the lesion (Figures 1A,B); (b) the patients still exhibited a high fever, chills and other symptoms, and the inflammation did not decrease as indicated, for example, by the failure of C-reactive protein (CRP) levels or decrease in white blood cell counts.

Nuclear magnetic resonance (NMR)-based metabonomic analysis is a systems biology approach that aims to detect global metabolic information in biological samples. This approach is widely used in infectious disease diagnosis and the study of pathogenesis (Chen et al., 2014; Embade et al., 2016; Li et al., 2017).

Therefore, the purpose of this study was to retrospectively compare the metabolic characterizations of host responses to DRKPLAs and drainage-sensitive Klebsiella pneumoniae liver abscesses (DSKPLAs; Figures 1C,D) with serum 1H-NMR spectroscopy. Our results demonstrated that statistical analyses of serum 1H-NMR spectra can effectively distinguish between these two groups of KPLA patients and thus provide a novel application of NMR spectroscopy.

## Materials and methods

### Patients and data

All serum samples were collected prospectively in a previous study [Title:Predictors from Clinical and CT features for metastatic infection among Patients with Klebsiella pneumoniae Liver Abscess, Registration at the Chinese clinical trial center (http://www.chictr.org.cn) with number:ChiCTR-ROC-15006581. The project was approved by the ethics committee of the Shengjing Hospital (Approved Number:2015PS184K), all patients provided written informed consent]. Shengjing hospital granted a waiver for this retrospective study.

Using our institutional electronic medical database, we retrieved the records of patients with a main diagnosis of a liver abscess between June 2015 and December 2016. The database search yielded 218 patients.

The inclusion criteria were as follows: (a) the presence of a focal lesion or lesions in the liver on contrast-enhanced CT images, (b) the receipt of percutaneous drainage therapy and the presence of frank pus aspirated from the abscess cavity through this procedure, and (c) positive microbiological culture results from the liver abscess and/or blood cultures in addition to culture results that indicated the presence of mono-microbial *K. pneumoniae*.

The medical records were reviewed for the following information: (a) demographic data; (b) coexisting medical conditions, including diabetes mellitus, biliary tract disease, malignancy, and cirrhosis; (c) clinical symptoms; (d) initial laboratory data, including the total and differential white blood cell counts, platelet counts, glucose, C-reactive protein and immune function (lymphocyte subsets); and (e) whether the patients underwent a surgical intervention.

### Lymphocyte subsets by flow cytometry

Flow cytometric enumeration of T, B, and NK cells were performed on FACSCalibur (BD Biosciences, San Jose, CA, USA) and the different cell populations were analyzed using the Cell Quest Pro software (v5.2). The monoclonal antibodies used to identify these cell subsets were as follows: anti-CD45, anti-CD3, anti-CD4, anti-CD8, anti-CD19, anti-CD16, anti-CD56, anti-CD45RA, and anti-CD45RO. Briefly, whole blood (100 μL) was labeled with respective antibodies (BD biosciences, USA) as per manufacturer's instructions. After 30 min incubation in dark at room temperature with mAbs, RBC's were lysed using FACS lysing solution and washed with phosphate buffered saline. Labeled lymphocytes were acquired on a FACSCalibur flow cytometer (BD Biosciences, San Jose, CA, USA) after proper instrument setting, calibration, and compensation. Absolute counts (Abs) for each cell subset were calculated by multiplying the specific subset percentages to absolute lymphocyte counts/100. The lymphocytes were gated as T cells (CD3+, CD3+CD8+, CD3+CD4+, CD4+CD45RA+, and CD4+CD45RO+), B cells (CD19+), and NK cells (CD16+CD56+).

### *K. pneumoniae* string test

The hypermucoviscous phenotype of *K. pneumoniae* can be identified by a positive string test. A bacterial loop was touched to a suspect colony on an agar plate and withdrawn slowly, and the bacteria that formed a mucoid “string” of 5 mm were considered to be positive (Wang et al., 2013).

### CT characteristics

All patients underwent contrast-enhanced CT prior to the drainage of the liver abscesses. If the clinical symptoms were not alleviated 3 days after drainage, or the quantity of drainage was very small, liver CT studies were also performed to assess the size of the abscess cavity and to determine the position of the drainage catheter in cases of poor or incomplete drainage or to monitor complications. Both CT examinations were performed, and the scans were reviewed as previously reported (Chang et al., 2015, 2016).

### Treatment

In addition to the percutaneous drainage therapy, all patients received antibiotic therapy (most commonly third-generation cephalosporins, ciprofloxacin, or carbapenem antibiotics) for their abscesses.

### ^1^H-NMR spectra

For the 1H-NMR analyses, 500 μL serum samples were thawed at a temperature of 4°C and then passed through a 3-KDa ultrafiltration filter (Millipore, USA). The filtered units were centrifuged at 13,000 rpm for 30 min at 4°C. The filtrates were collected and mixed with 50 μL Anachro Certified DSS Standard Solution (ACDSS), vortexed (10 s) and centrifuged (13,000 rpm, 2 min, 4°C). The prepared NMR samples were loaded into 5-mm NMR tubes. The 1H-NMR measurements were performed at 298.15 K on an Agilent 600 MHz spectrometer equipped with a triple-resonance cryoprobe (Agilent Technologies, USA) that was operated at 599.83 MHz to examine the 1H resonance frequency. A METNOESYpulse sequence was used to determine the serum metabolite profiles (Weljie et al., 2006). Sixty-four transitions were collected with a frequency domain size of 65,536 Hz. The relaxation delay time was set to 1 s, which matched the spectra acquisition condition of the Chenomx reference standard database. The 1H-NMR free induction decay signal was imported into the Chenomx NMR suite version 8.0 (Chenomx, Canada), and the data were automatically Fourier transformed and phase-adjusted. The baseline was carefully adjusted by experienced technicians inside the processor module. Metabolites from the serum were qualified and quantified using the Chenomx NMR Suite software package (Tredwell et al., 2011). Briefly, the qualification was performed by matching the sample spectra against the spectra of a pH-adjusted standard reference inside the Chenomx Database. The quantification was performed by comparing the area under the deconvoluted peak against the peak area of the 2,2-dimethyl-2-silapentane-5-sulfonate sodium salt (DSS) signal at 0 ppm.

Partial least square discriminant analysis (PLS-DA) was applied to analyze the spectral data and separate the DRKPLA from the DSKPLA samples. Loading plots were used to identify the spectral variables that were responsible for sample separation in the corresponding score plots. The variable importances in the projections (VIPs) of all peaks from the PLS-DA models were taken as the coefficients for metabolite selection, and the variables with VIP values >1 were considered to be contributors to the group discrimination (Afanador et al., 2014). Student's *t*-test (*p* < 0.05) was also used to detect significant differences in metabolites between the DRKPLA and DSKPLA samples using Metaboanalyst ver 3.0 (http://www.metaboanalyst.ca/MetaboAnalyst/). Only the variables with VIP values >1 and *p* values < 0.05 were considered significant.

### Pathway analysis

Metaboanalyst version 3.0 was used to perform the pathway analysis and to visualize all of the chemical metabolites that were present at different levels in the serum samples from the DSKPLA and DRKPLA patients (Xia et al., 2015).

### Statistical analysis

Data about the subjects were analyzed using SPSS, version 17.0 (SPSS Inc.). Normally distributed continuous variables are presented as the means ± the SDs and were compared using Student's *t*-test. Categorical variables were compared using Fisher's exact test. All tests were two-sided, and *p* < 0.05 was considered statistically significant.

## Results

### Clinical features

**Figure 2:**
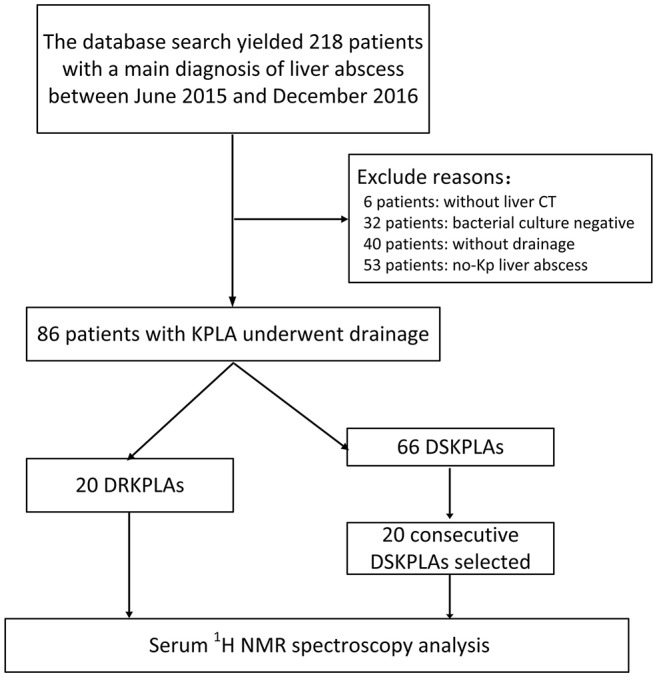
Flow chart of the patient selection.

In total, 86 patients with *K. pneumoniae* liver abscesses who underwent percutaneous drainage were identified. The patient selection process is illustrated in in Figure 2. Twenty patients (23.3%) had DRKPLAs, and 66 patients (76.7%) had DSKPLAs. Comparisons of the demographic data, laboratory findings, and other clinical features of the patients with DRKPLAs and DSKPLAs are presented in Table 1. The DRKPLA group has a significantly higher rate of diabetes compare to DSKPLA group (70% vs. 51.5, *p* = 0.03). The *K. pneumoniae* strain causing infection in the DRKPLA group is more likely to be of the hyperviscous phenotype (80 vs. 46.9%, *p* = 0.01).The days of hospitalization in DRKPLA group is significantly longer than DSKPLA group (21.76 ± 12.38 vs. 8.04 ± 4.63 days, *p* < 0.01).

**Table 1:** Clinical and CT Characteristic in 86 patients with KPLA underwent drainage.

**Characteristic**	**DRKPLA (*n* = 20)**	**DSKPLA (*n* = 66)**	P*
Age (years)	52.44 ± 11.71	55.83 ± 10.97	0.12
Sex (male)	14 (70.0)	53 (80.3)	0.21
**UNDERLYING DISEASE**			
Diabetes mellitus	14 (70.0)	34 (51.5)	0.03
Cryptogenic	5 (25.0)	17 (25.7)	0.26
Biliary tract disease	1 (5.0)	6 (9.1)	0.32
**PRESENTING SYMPTOMS**			
Fever (≥38°C)	19 (95.0)	60 (90.9)	0.68
Abdominal pain/discomfort	17 (85.0)	51 (77.2)	0.43
Gastrointestinal symptoms	4 (20.0)	18 (27.3)	0.11
**HEMATOLOGICAL PARAMETERS**			
White blood cells (× 10^9^/L)	16.03 ± 5.59	14.56 ± 6.69	0.31
CRP (mg/L)	183.91 ± 143.74	225.47 ± 81.65	0.51
ALT	121.85 ± 74.69	90.60 ± 51.97	0.09
Total bilirubin	28.25 ± 13.20	30.94 ± 14.48	0.19
Septic Shock	2 (10.0)	5 (7.6)	0.30
Days of hospitalization	21.76 ± 12.38	8.04 ± 4.63	< 0.01
**CT CHARACTERISTIC**			
Maximal abscess diameter(mm)	62.87 ± 29.77	69.67 ± 34.58	0.06
Single	16 (80.0)	44 (66.7)	0.10
Solid	19 (95.0)	37 (56.1)	0.02
Multilocular	16 (80.0)	38 (57.6)	0.04
Gas formation	1 (5.0)	14 (21.2)	< 0.01
Hypermucoviscosity phenotype	17 (85.0)	31 (46.9)	0.01

Data are presented as median (mean ± standard deviation) or n (%). DRKPLA, drainage-resistant Klebsiella pneumoniae liver abscess; DSKPLA, drainage-sensitive Klebsiella pneumoniae liver abscesses.*DRKPLA vs. DSKPLA.

### CT characteristics

Comparisons of the CT characteristics of the DRKPLAs (Figures 1A,B) with the DSKPLAs (Figures 1C,D) are summarized in Table 1. The DSKPLAs predominantly exhibited solid CT appearances, were multilocular, and exhibited less gas formation (*p* < 0.05).

### Immune function analysis

Seven patients underwent immune function tests in this study. The CD16+CD56+NK cells were significantly decreased in the DRKPLA patients compared with the DSKPLA patients (*p* = 0.02). The analyses of the peripheral blood lymphocyte subsets are presented in Table 2.

**Table 2:** Analysis of peripheral blood lymphocyte subsets in seven patients.

**Items**	**The proportions of lymphocyte subsets %**		p*
	**DRKPLA(*n* = 3)**	**DSKPLA(*n* = 4)**	
**T CELL SURFACE ANTIGENS**			
**CD3+**	**85.04 ± 5.84**	**84.22 ± 2.42**	**0.81**
**CD3+CD8+**	**39.89 ± 16.00**	**33.78 ± 20.66**	**0.69**
**CD3+CD4+**	**33.76 ± 27.58**	**48.62 ± 19.12**	**0.43**
**CD4+CD45RA+**	**29.08 ± 7.14**	**52.75 ± 28.00**	**0.22**
**CD4+CD45RO+**	**19.33 ± 1.32**	**48.12 ± 20.14**	**0.06**
**B CELL SURFACE ANTIGENS**			
**CD19+**	**8.77 ± 6.72**	**6.45 ± 3.74**	**0.58**
**NK CELL SURFACE ANTIGENS**			
**CD16+CD56+**	**4.76 ± 1.20**	**8.12 ± 1.21**	**0.02**

Data are presented as median (mean ± standard deviation). DRKPLA, drainage-resistant Klebsiella pneumoniae liver abscess; DSKPLA, drainage-sensitive Klebsiella pneumoniae liver abscesses.*DRKPLA vs. DSKPLA.

### Characteristics of the serum sample population

We collected a total of 40 serum samples, including 20 DRKPLAS and 20 DSKPLAs (to reduce costs, we only selected 20 consecutive patients as the controls), which were included in the metabolomics study. The demographic information about these patients is summarized in Table 3. The DRKPLA group has only a higher rate of diabetes compare to DSKPLA group (70 vs. 45%, *p* = 0.01) in the study of serum metabolomics. Other consistent characteristics reduce the influences of differences in individual conditions and ensure that the findings are attributable to the two disease conditions.

**Table 3:** Characteristics of the serum sample population.

**Characteristic**	**DRKPLA (*n* = 20)**	**DSKPLA (*n* = 20)**	P*
Age (years)	52.44 ± 11.71	54.63 ± 11.29	0.15
Sex (male)	14 (70.0)	15 (75.0)	0.28
BMI	23.49 ± 2.49	22.3 ± 2.17	0.36
**COMBINED DISEASE**			
Diabetes mellitus	14 (70.0)	9 (45.0)	0.01
Hypertension	5 (25.0)	4 (20.0)	0.62
Hyperlipemia	6 (30.0)	7 (35.0)	0.52
Liver cirrhosis	1 (0.025)	0	0.29

Data are presented as median (mean ± standard deviation) or n (%). DRKPLA, drainage-resistant Klebsiella pneumoniae liver abscess; DSKPLA, drainage-sensitive Klebsiella pneumoniae liver abscesses.*DRKPLA vs. DSKPLA.

### Analysis of the serum ^1^H NMR spectra reveals distinct metabolic features

**Figure 3:**
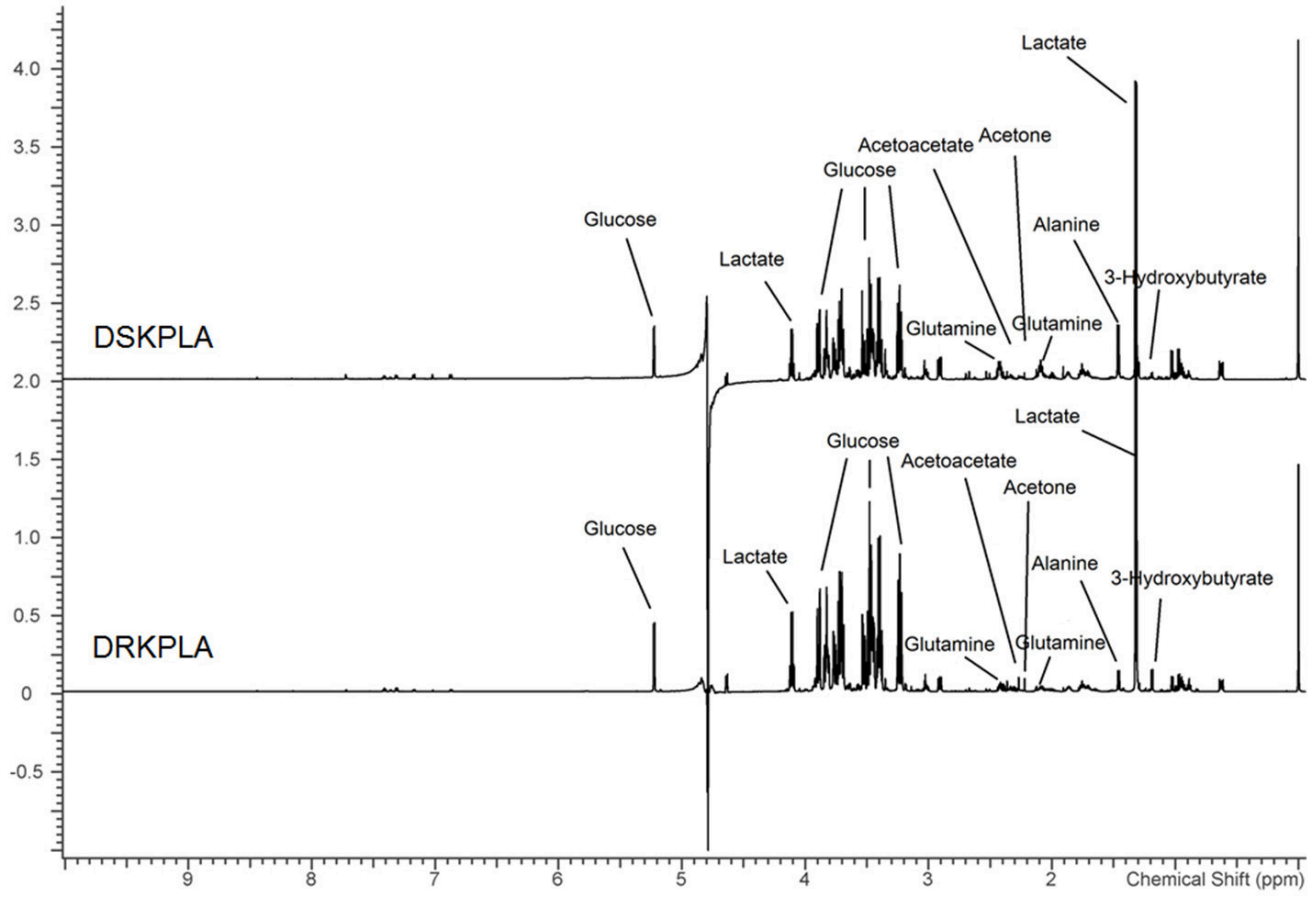
Representative 599.83 MHz 1HNMR spectra of serum samples from a DSKPLA and a DRKPLA patient. The key metabolites are noted.

**Figure 4:**
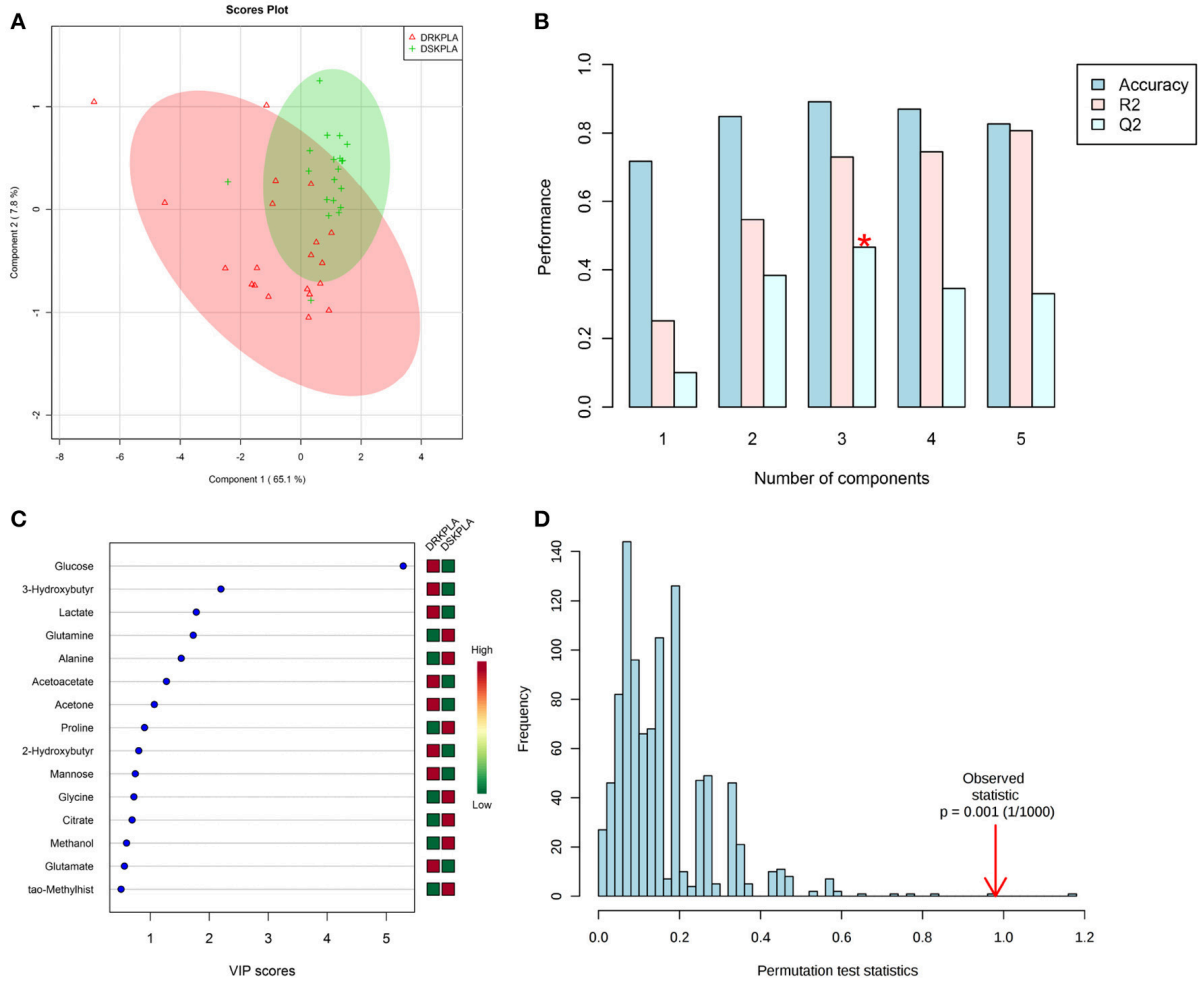
PLS-DA analyses of the DRKPLAs and the DSKPLAs serum samples. **(A)** Two-dimensional PLS-DA score plot. **(B)** PLS-DA classification using different numbers of components. The red asterisk indicates the best classifier. The inset table summarizes the *Q*^2^, *R*^2^ and accuracy of the best model. **(C)** Important features identified by the VIP scores. The variable importance in projection score is a weighted sum of squares of the PLS-DA loadings that accounts for the amount of explained Y-variation in each dimension. **(D)** Permutation test statistics for 1,000 permutations with the observed statistic at *p* < 0.01.

A series of changes in the endogenous metabolite levels was observed when the DRKPLA patients were compared with the DSKPLA patients. Typical spectra of the serum samples from a DRKPLA patient and a DSKPLA patient are presented in Figure 3. We used PLS-DA to visualize the metabolic differences between these two patients. There were separate trends in both groups in the PLS-DA score plots (Figure 4A), which indicated that they had different metabolic characteristics. To evaluate the statistical robustness of the analysis, a 10-fold cross-validation was performed, and the *Q*^2^ and *R*^2^ values were deduced. Our model provided high *R*^2^ and *Q*^2^ scores, which confirmed a good predictive power (Figure 4B). Figure 4C illustrates the key differentiating features that were identified by the PLS-DA analysis sorted by increasing VIP score. Permutation tests (1,000 repeats) yielded a very low *p*-value (< 0.01), which indicated that none of the distributions formed by the permuted data were better than the observed statistic based on the original data (Figure 4D).

**Figure 5:**
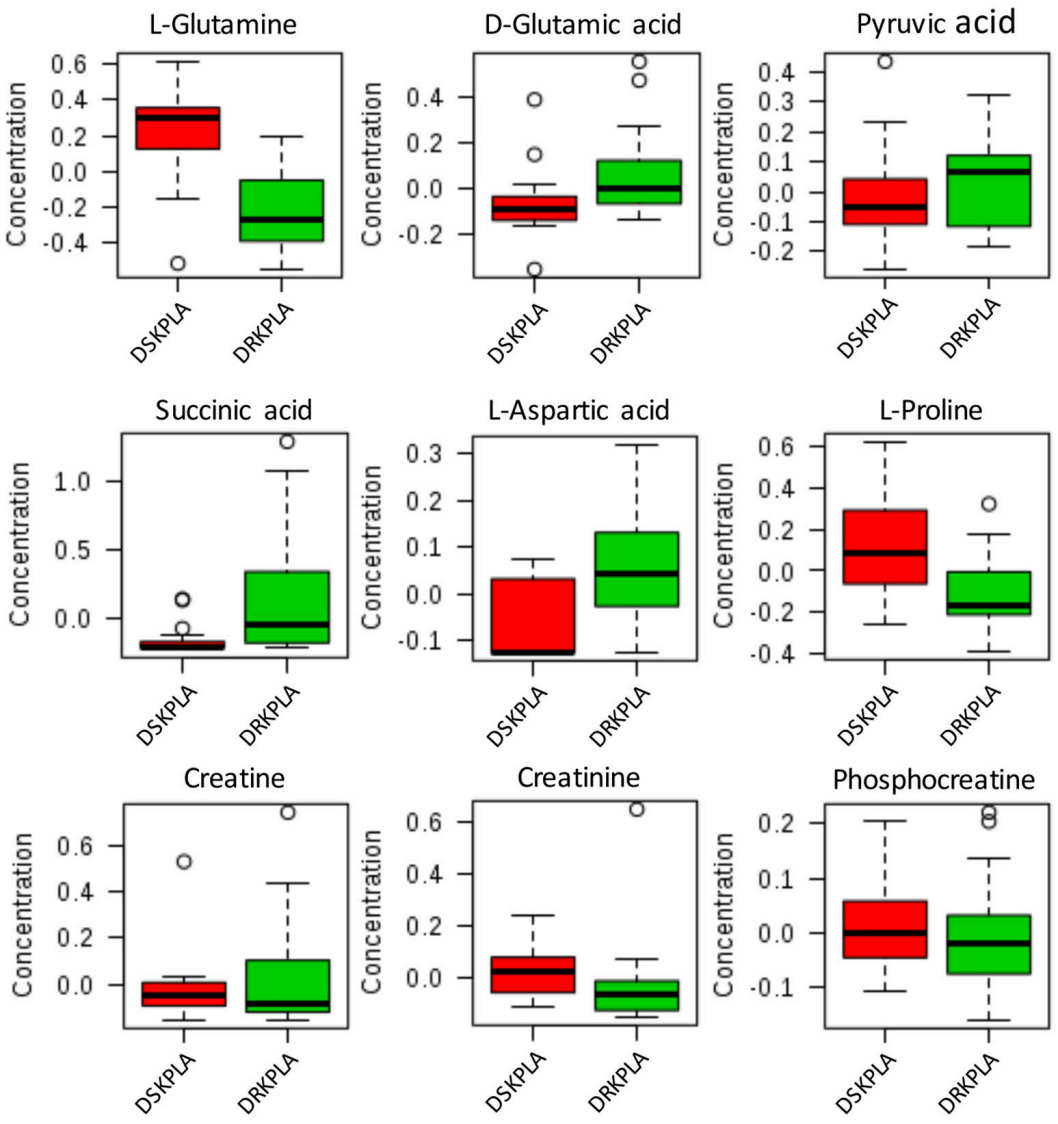
Boxplot of the relative concentrations of the significantly altered metabolites (*p* < 0.05) in the sera of DRKPLA (green) and DSKPLA (red) patients. The bar plots show the normalized values (the mean ± one standard deviation). The boxes range from the 25% to the 75% percentiles, and the 5% and 95% percentiles are indicated as error bars. Single data points are indicated by circles. The medians are indicated by the horizontal lines within each box.

Five metabolites that could be used to discriminate between DRKPLAs and DsKPLAs were identified based on chemical shifts (VIP >1 and *p* < 0.05). Glucose, lactate and 3-hydroxybutyrate were found to be upregulated in the DRKPLAs, and glutamine and alanine were downregulated in the DRKPLAs compared with the DSKPLAs. Figure 5 provides some example boxplots of metabolites that were significantly different in the serum of the DRKPLAs and DSKPLAs patients. All metabolites concentration is provided in Supplementary Table 1.

### Pathway analysis

**Figure 6:**
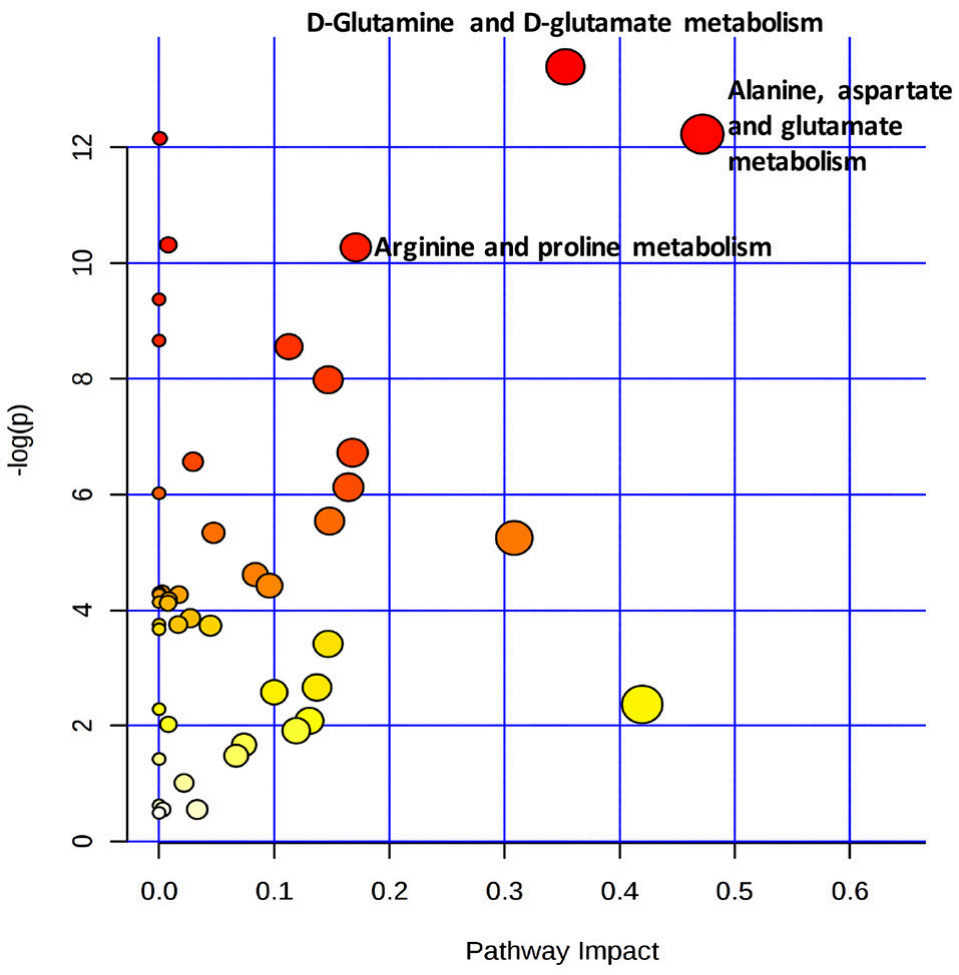
Metabolic pathway analysis of the key metabolites present in the serum samples. The pathways named in the figure had impact values >0.15 and –log(p) values >10.

With A combination of an pathway impact>0.15 and –log(p) >10, the results of the pathway analysis revealed that the three most strongly influence canonical pathways were alanine, aspartate and glutamate metabolism, D-glutamine and D-glutamate metabolism, and arginine and proline metabolism (Figure 6 and Table 4).

**Table 4:** Pathway analysis of key metabolites.

**Pathway name**	**Hits^a^**	***p***	**–Log(p)**	**FDR^b^**	**Impact^c^**
Alanine, aspartate and glutamate metabolism	2	1.53E-06	13.392	7.49E-05	0.35294
D-Glutamine and D-glutamate metabolism	4	4.89E-06	12.228	8.61E-05	0.47199
Arginine and proline metabolism	8	3.46E-05	10.271	3.39E-04	0.17075
Butanoate metabolism	4	0.003915	5.543	0.011989	0.148
Purine metabolism	3	3.31E-05	10.317	3.39E-04	0.00794
Nitrogen metabolism	6	5.27E-06	12.154	8.61E-05	6.70E-04
Aminoacyl-tRNA biosynthesis	10	1.92E-04	8.5565	0.001178	0.11268
Glyoxylate and dicarboxylate metabolism	4	3.41E-04	7.9839	0.001856	0.14685
Citrate cycle (TCA cycle)	3	0.001201	6.725	0.005883	0.005883
Fructose and mannose metabolism	1	0.001404	6.5682	0.006256	0.02948
Methane metabolism	5	0.002181	6.128	0.008475	0.16439
Synthesis and degradation of ketone bodies	3	0.003696	5.6006	0.011989	0.7
Tyrosine metabolism	4	0.004791	5.341	0.01381	0.01381
Glycine, serine, and threonine metabolism	7	0.005242	5.2511	0.014269	0.30843
Valine, leucine, and isoleucine degradation	5	0.009874	4.6178	0.025465	0.0835
Glycolysis or Gluconeogenesis	5	0.011955	4.4266	0.028519	0.09576
Galactose metabolism	3	0.013393	4.313	0.028519	0.00276
Starch and sucrose metabolism	1	0.013969	4.2709	0.028519	0.01703
Primary bile acid biosynthesis	1	0.015259	4.1826	0.029381	0.00822
Glutathione metabolism	2	0.01619	4.1234	0.029381	0.00762
Vitamin B6 metabolism	2	0.020978	3.8643	0.036712	0.02712
Cysteine and methionine metabolism	2	0.023407	3.7547	0.037672	0.01649
Propanoate metabolism	6	0.023833	3.7367	0.037672	0.04451
Lysine degradation	3	0.032891	3.4146	0.048838	0.14675
Inositol phosphate metabolism	1	0.070177	2.6567	0.10114	0.13703
Lysine biosynthesis	2	0.076268	0.076268	0.10677	0.09993
Pyruvate metabolism	4	0.094084	2.3636	0.12806	0.41957
Ascorbate and aldarate metabolism	3	0.12483	2.0808	0.16097	0.13047
Phenylalanine metabolism	4	0.14801	1.9105	0.18131	0.11906
Valine, leucine, and isoleucine biosynthesis	5	0.18881	1.667	0.22565	0.07367
Glycerophospholipid metabolism	2	0.22803	1.4783	0.26604	0.06691
Taurine and hypotaurine metabolism	2	0.36489	1.0082	0.39733	0.02158
Selenoamino acid metabolism	1	0.57638	0.55098	0.58839	0.00321

a *Hits, the number of compounds that match with our experimental data*;

b *FDR, False Discovery Rate*;

c *Impact, pathway impact value calculated from pathway topology analysis*.

## Discussion

In addition to effective antimicrobial therapy, percutaneous drainage is recognized as the most safe and effective treatment for PLA because this treatment can reduce the pressure inside the abscess and lower the bacterial load (Liu et al., 2009; Liao et al., 2012). The effectiveness of drainage depends on the extent of liquefaction of the abscess and whether it is multilocular (Hui et al., 2007; Liao et al., 2012). Therefore, it is much more difficult to drain KPLAs that are characterized as solid or multiloculated. The data from the present study further strengthen the evidence that KPLAs with solid or multilocular appearances are associated with difficult drainage.

Our study results revealed that the hypermucoviscous strain of *K. pneumoniae* may be one of the causes of drainage resistance. Sticky pus can block the drainage tube or slow down the process of drainage. The high level of virulence observed in hypervirulent *K. pneumoniae* strains is due to an increased expression of capsular material that is related to the hypermucoviscous phenotype (Keynan et al., 2007; Wang et al., 2013). The factor that mediates the expression of the hypermucoviscous phenotype is RmpA/RmpA2 (Chuang et al., 2013; Qu et al., 2015). Because we don't have microbiology experiment conditions, we could not detect the serum types and virulence genes of *K. pneumoniae*. The other possible explanation for the solid appearance of KPLAs is the failure of liquefaction due to a high prevalence of a phagocytosis-resistant capsular serotype *K. pneumoniae* strain (Hui et al., 2007; Alsaif et al., 2011). However, thus far, there is no experimental evidence to support this notion. However, we found that CD16+CD56+ natural killer (NK) cells were significantly decreased in the DRKPL compared with the DSKPLAs via an analysis of the peripheral blood lymphocyte subsets. NK cells are innate lymphocytes that play important roles in the defense against microbial pathogens through the secretion of IFN-γ and recognition and lysis of virally or bacterially infected host cells (Fuchs and Colonna, 2011). On the other hand, diabetes can weaken the phagocytosis of macrophage and impaired host immunity. Both the engulfing and digesting of bacteria by host cells and the lysis of bacterial-infected host cell are associated with the liquefaction of abscesses.

To further explore the host response mechanisms, we used ^1^H NMR-based metabonomic analysis with the aim of detecting global metabolic information from the sera of DRKPLA patients and comparing the results to those from DSKPLA patients. We found that the discrimination between the two patient groups was indeed possible via comparisons of the concentrations of metabolites. Glucose was found to be upregulated in DRKPLAs. Patients with diabetes are at an increased risk of KPLA due to impaired host immunity, poor blood supply, nerve damage, and alternations in metabolism (Wang et al., 2014). Thus, strict control of glucose during the treatment of an abscess is very important. In addition to glucose, four key metabolites identified in our study may be potential targets for promoting the liquidation of DRKPLAs, i.e., lactate, 3-hydroxybutyrate, glutamine and alanine. However, there is little information available from related studies with which to compare this finding. Chen et al. found different metabolic profiles of K1 serotype and non-serotype K1 and K2 Klebsiella pneumoniae isolates in a mouse model of oral infection, but these authors did not study the relationships of the serotype with abscess liquefaction or the prognosis of puncture drainage (Chen et al., 2014). The K1/K2 capsular polysaccharide (CPS) has proven to be significantly more resistant to phagocytosis than the non-K1/K2 CPS in liver abscess isolates (Lin et al., 2004; Yeh et al., 2006). The pathway analysis in our study revealed that alanine, aspartate and glutamate metabolism, D-glutamine and D-glutamate metabolism, and arginine and proline metabolism were the three most influential canonical KEGG pathways, which suggests that there is are significant differences in the amino acid profiles of sera from DRKPLA and DSKPLA patients. Dietary glutamine supplementation could partly reverse the impaired macrophage function and could increase NK cell activity and reduce the immunosuppressive effects that result from overload training (Song et al., 2015; Xiao et al., 2015). Therefore, we hypothesized that supplementation with glutamine may improve macrophage phagocytosis and the host immune defense against *K. pneumoniae* and thus promote abscess liquefaction. Animal experiments are expected to verify our hypothesis in the future.

Our study had limitations. First, it was retrospective in design. Second, in the serum metabolic analysis, only 20 cases were included as controls, which might have led to potential bias. Third, because the serum samples were obtained after the patients were diagnosed with liver abscesses, we cannot entirely rule out the notion that drainage resistance led to increased inflammation and then affected the metabolic changes. However, regardless of causality, the goal of this study was simply to identify potential targets for promoting the liquefaction of DRKPLAs.

In conclusion, our study results demonstrate that glucose and the other four key identified metabolites may be potential targets for guiding novel therapeutics of DRKPLAs and are worthy of additional investigation.

## Author contributions

ZC, HW, BL, and ZL designed and conduct the study, analyzed the data, and draft the manuscript. JZ and ZL supervised the study. All authors read and approved the final manuscript.

### Conflict of interest statement

The authors declare that the research was conducted in the absence of any commercial or financial relationships that could be construed as a potential conflict of interest.

## Supplementary material

Supplementary Table 1 The concentration of different metabolites in plasma in both groups and the name of metabolites involved in the pathway analysis.
